# Queer Genes: Realism, Sexuality and Science

**DOI:** 10.1080/14767430.2016.1210872

**Published:** 2016-08-23

**Authors:** David Andrew Griffiths

**Affiliations:** ^a^ University of Surrey, Guildford, UK

**Keywords:** agential realism, critical realism, feminist standpoint epistemologies, gay genes, queer, situated knowledges

## Abstract

What are ‘gay genes’ and are they real? This article looks at key research into these hypothesized gay genes, made possible, in part, by the Human Genome Project. I argue that the complexity of both genetics and human sexuality demands a truly critical approach: one that takes into account feminist epistemologies of science and queer approaches to the body, while putting into conversation resources from agential realism and critical realism. This approach is able to maintain the agential complexity of genetic materiality, while also critically challenging the seemingly stable relationships between sex, gender and sexuality.

One can start from the idea that the world is filled not, in the first instance, with facts and observations, but with *agency*. The world … is continually *doing things*, things that bear upon us not as observation statements upon disembodied intellects but as forces upon material beings. (Pickering 1995, 6)

What are ‘gay genes’, and are they real? Such questions are clearly complex in nature, and seem to require at least working definitions of the terms ‘gay’, ‘genes’ and of course ‘reality’. In this article, I will bring into dialogue critical realist, feminist science studies, and queer approaches to issues of science and sexuality in order to develop new resources for thinking about the complex issue of gay genes. First, I will discuss key pieces of research into a genetic basis for homosexuality. I will focus on two studies in particular, published in the early 1990s. Subsequent work has been published in this area but, if the twentieth century is the century of the gene (Keller 2000), then I take the 1990s to be the decade of the genome. Throughout these sections I will argue that the process of developing social and scientific definitions of genes occurs within a number of co-acting and historically and socially situated causal contexts, including particularly the multi-billion dollar, multi-national Human Genome Project. In addition, I will argue that both strong constructivist and naïve scientific realist approaches to gay genes are insufficiently nuanced, as these approaches play up the importance of the biological at the expense of the social (or vice versa) as opposed to seeing the biological and the social as complexly co-forming.

I argue that what is needed, instead, is an approach that takes seriously the material and agential nature of genes, but also recognizes the fallible and situated (and therefore cultural, social and political) nature of all knowledge claims about these genes. In order to develop such an approach I will draw on both critical realism and various feminist approaches to scientific knowledge and reality. It should be noted, however, that despite the fact that these approaches have some shared concerns, there are a number of tensions that must be navigated when bringing them into conversation in this way. In this article I contend, however, that bringing these approaches into conversation — even without resolving all of the tensions between them — is a useful exercise to engage in. This is the case, in particular, because these approaches all attempt to negotiate between the poles of strong social constructivism and naïve scientific realism and therefore allow us to develop a new reading of the scientific and popular discourses around gay genes. Indeed, drawing on these approaches allows me to develop an approach to the reality of (gay) genes that is serious about materiality, as well as the situatedness and fallibility of all claims to knowledge.

## Genes and gay genes

In the early 1990s, two particularly influential pieces of scientific research into the genetic basis of sexual orientation were published: J. Michael Bailey and Richard Pillard's twin study (1991), and Dean Hamer et al.'s study of so-called genetic markers for homosexuality on the X chromosome (1993).^1^ These studies were made possible by the Human Genome Project. This project began in the late 1980s, and was supported by the United States Department of Energy. Historian of Science Daniel J. Kevles (1992, 18) suggests that much of the early impetus of the project came from initiatives that were undertaken during the 1980s by the molecular biologist Robert Sinsheimer and the physicist and mathematical biologist Charles DeLisi. These two scientists organized workshops on the prospects for engaging in a Human Genome Project, most notably in Santa Cruz in 1985 and Los Alamos in 1986. It was during these workshops, importantly, that the scale of, and some of the technical approaches to, a human genome project were outlined. Indeed, it was during these meetings that Gilbert persuaded a number of key scientists of the merit of engaging in such a venture, including Nobel laureate James D. Watson, co-discoverer of the helical structure of DNA. In the late 1980s, the US National Institutes of Health (NIH) then proceeded to endorse the Department of Energy-backed Human Genome Project, and Watson agreed to head the NIH office of the Human Genome Project in 1988. Finally, the project was officially inaugurated as a federal programme in 1991, and became a multi-billion dollar, multi-national project, worked on in both private and public laboratories across Europe and North America.

Importantly, these workshops also set the tone for the language that would be used during the project. It was at the Los Alamos meeting in March 1986, for instance, that Walter Gilbert, molecular biologist and Nobel Laureate, described the human genome as the Holy Grail of biological research (Gilbert 1992; see also Lewontin 2000). Such turns of phrase were typical features of the early stages of the research; this was a period characterized by optimism and exuberance especially for key figures such as Gilbert and Watson. The project was commonly described in terms of mapping the human genome, or reading the book of life (Rosner and Johnson 1995).^2^ In ‘A Personal View of the Project’, furthermore, Watson describes the aim as ‘a complete genetic blueprint of man’ (Watson 1992, 164), and suggests setting up a databank of overlapping genetic markers, which he imagines as a ‘library for the entire human genome’ (169). While, in places, Watson refers to the genome sequence as a ‘blueprint’, ‘description’ and ‘map’, elsewhere he refers to the project as being able to explain ‘how life works’ (164–7), and he predicts the possibility of not just *describing* the human genetic sequence but of ‘*knowing* the human genome’ (173, emphasis added). His account is therefore arguably illustrative of a fairly naïve form of scientific realism, as he claims that the descriptions and maps that are going to be produced by the project will be able to faithfully and unproblematically represent the biological real. For Watson, in short, the draft of the human genome allows access to the truth of human biology and life.

This hubristic level of optimism with regard to genetic research was also shared by the two aforementioned studies into the genetic basis of sexual orientation. The first of these studies, Bailey and Pillard's ‘A Genetic Study of Male Sexual Orientation’, was published in 1991.^3^ In order to construct this study the authors advertised in magazines for gay men with a twin brother, or an adoptive brother who had been adopted into the family at an age of less than three. These gay men were then interviewed about their sexuality and the sexualities of their twin, or adoptive, brothers, and were sent questionnaires to determine their sexuality. The twin brothers were also asked to confirm if they were monozygotic (identical — from a single egg) or dizygotic (non-identical — from two eggs) twins. Of the participants included in the final sample, ‘52% (29/56) of monozygotic cotwins, 22% (12/54) of dizygotic cotwins, and 11% (6/57) of adoptive brothers were homosexual’ (Bailey and Pillard 1991, 1089). These results led Bailey and Pillard to conclude that there is a genetic basis for homosexuality. Pillard, in fact, took this claim further: in an interview in the journal *GLQ* he stated, ‘we think that the hypothesized gay genes are real’ (Stein 1991, 94).

It takes something of a leap of faith, however, to think that gay genes are real, based on this evidence. Many critics have suggested that the numbers simply do not support this genetic conclusion. William Byne and Bruce Parsons, for instance, state that:

the concordance rate for homosexuality in nontwin biologic brothers was only 9.2% — significantly lower than that required by simple genetic hypothesis, which, on the basis of shared genetic material, would predict similar concordance rates for [dizygotic] twins and nontwin biologic brothers. Furthermore, the fact that the concordance rates were similar for nontwin biologic brothers (9.2%) and genetically unrelated adoptive brothers (11.0%) is at odds with a simple genetic hypothesis, which would predict a higher concordance rate for biological siblings. (1993, 229; see also Hubbard and Wald [1993] 1999; Byne 1994)

Monozygotic twins, after all, share almost identical DNA, yet close to half of the sampled monozygotic twins did not share a sexual orientation. Despite this, Pillard interprets the results as proof not only of a genetic basis for homosexuality, but as suggesting the existence of a specific gene — or genes — that code for homosexuality.

Two years later Dean Hamer et al. published ‘A Linkage etween DNA Markers on the X Chromosome and Male Sexual Orientation’ (1993). Following on from previous work on the biological basis of homosexuality, Hamer took advantage of the developments that the massive multinational funding of the Human Genome Project had made possible, stating that:

Recent advances in human genome analysis, in particular the development of chromosomal genetic maps that are densely populated with highly polymorphic markers, make it feasible to apply such methods to complex traits, such as sexual orientation, even if these traits are influenced by multiple genes or environmental or experiential factors, or some combination of these. (1993, 321)

The subjects of Hamer's study were self-identified gay men and their relatives over the age of 18. The final sample consisted of 38 pairs of homosexual brothers and their relatives, with two families added from a previous sample. The total participants numbered 114. The 38 families were chosen very deliberately, in order to test the hypothesis that there is a maternally transmitted genetic basis for homosexuality (Hamer et al. 1993, 322). The sample demonstrated a high number of homosexual maternal uncles and sons of maternal aunts, something that confirmed the findings of previous research. Following this, Hamer looked at the X chromosome for possible maternally transmitted genetic markers for homosexuality.^4^ He reported that, by means of chromosome mapping, significant correlations were observed; in particular, he identified links between genetic marker Xq28 and self-reported homosexuality in his sample. Hamer therefore concluded that:

We have now produced evidence that one form of male homosexuality is preferentially transmitted through the maternal side and is genetically linked to chromosomal region Xq28 … it appears that Xq28 contains a gene that contributes to homosexual orientation in males. (Hamer et al. 1993, 325)

As with Bailey and Pillard's twin studies, however, there are numerous methodological problems with Hamer's research. Hamer's sample is limited, for instance, and there are issues of sampling bias, as the research focused on a specific group of gay men with a particular family background. In addition, not only was the focus on a specific group of gay men, but an adequate control group (such as nonhomosexual brothers) was not included (see Fausto-Sterling and Balaban 1993). Additional research attempting to confirm or replicate these findings has also produced ambiguous results.^5^ Hamer's tone in the *Science* article is, however, appropriately speculative and specific. He states that the subject is complex, that a single genetic locus cannot account for the variability of human sexuality (Hamer et al. 1993, 325–6), and that his study has only shown that ‘*one form of homosexuality* is … genetically linked to chromosomal region Xq28’ (325, emphasis added). However, in his popular science book, *The Science of Desire: The Search for the Gay Gene and the Biology of Behavior*, Hamer states, more forcefully, that although ‘we didn't isolate a “gay gene”’ we did detect ‘its presence through linkage’ (Hamer and Copeland 1994, 147).

## What are genes anyway?

In addition to displaying this kind of optimism about their specific results, however, it is also worth noting the broader point that, for all of the researchers involved in the aforementioned studies, the ‘reality’ of the gay gene (or genes) is not in question. Although their research may not in fact have unambiguously identified a gay gene, its existence is assumed to be self-evident and is held to be supported by their respective study. Indeed, in both studies, genes are simply assumed to be (although they are not always explicitly defined as) a structural biological unit, a molecule that in some way determines behaviour or identity. Such views are not necessarily supported, however, by the broader literature on this topic. For instance, at an historical level, the word ‘gene’ did not always have such a deterministic meaning. In fact, the term was coined in 1909 by botanist Wilhelm Johannsen, who simply wanted to develop a word that could refer to the evident fact that characteristics were transmitted across generations. As he stated:

The word ‘gene’ is completely free from any hypothesis; it expresses only the evident fact that, in any case, many characteristics of the organism are specified in the gametes by means of special conditions, foundations, and determiners which are present in unique, separate, and thereby independent ways — in short, precisely what we wish to call genes. (Johannsen 1909, 124, quoted and translated in Keller 2000, 2)

Johannsen underscored this point again two years later, stating that: ‘The “gene” is nothing but a very applicable little word’ (1911, 132–4). Despite Johannsen's desire to keep genes free from any hypothesis, however, Evelyn Fox Keller argues that, by the 1930s, genes had ‘become incontrovertibly real, material entities — the biological analogue of the molecules and atoms of physical science’ (2000, 2). Proof for the reality and materiality of genes seemed to materialize in 1953, with the discovery of the function and form of DNA. Following this, the classical view of the gene became that it was a string of DNA that, when translated into messenger RNA (mRNA), would produce a protein. This picture of the gene, importantly, fit neatly with the neo-Darwinian narrative of evolution: DNA is a molecule that occasionally mutates, producing morphological differences due to the difference in proteins, and evolution proceeds by a cumulative selection of the mutations that result in increased fitness (Keller 2000, 35). Whatever the truth of such claims, Keller states that, ‘by midcentury, all remaining doubts about the material reality of the gene were dispelled and the way was cleared for the gene to become the foundational concept capable of unifying all biology’ (2000, 3). Not only this, but the way was cleared for the gene to function, in what Keller calls ‘gene talk’, as a ‘master molecule’ — a notion that, far from being free from hypothesis, functions rhetorically to support various hypotheses about the plausibility of biological determinism (2000, 133–48). One unexpected consequence that was generated by the Human Genome Project, however, was the realization that such an understanding was in fact overly simplistic. Keller, for instance, provides a list of some of the discoveries that have troubled the classical picture of the gene:

Techniques and data from sequence analysis have led to the identification not only of split genes but also of repeated genes, overlapping genes, cryptic DNA, antisense transcription, nested genes, and multiple promotes (allowing transcription to be initiated at alternative sites and according to variable criteria). All of these variations immeasurably confound the task of defining the gene as a structural unit. (2000, 67)

Some geneticists have suggested, in response to such complexity, that the word is no longer useful. William Gelbart, for instance, has suggested that the word is of limited value, and that it may even hinder genomic understanding and scientific progress (1998, 660). This is the case, he argues, because the word gene, although useful for scientists working in specific fields, does not necessarily refer to a single, stable biological entity, and thus cannot be guaranteed to have the same meaning across more than one sub-discipline of genetics. As Gelbart states, he finds it ‘very difficult to tell what someone means when they talk about genes because we don't share the same definition’ (Pearson 2006, 401). Francis Collins, director of the National Human Genome Research Institute (a role previously held by James Watson), has supported this view of the gene as well, stating that ‘we almost have to add an adjective every time we use that noun’ (Pearson 2006, 401). The applicability of Johannsen's little word is, therefore, not without its problems; ambiguously applied to different biological entities and processes it may, surprisingly, serve to undermine rather than support various reductive narratives of genetic determinism.

Despite such difficulties and ambiguities, however, this article will not argue in favour of abandoning the term. Rather, it will explore the possibility of thinking about genes (and ‘gay genes’) as real material biological and social entities without ignoring these ambiguities. In particular, it will suggest that — by drawing on a variety of feminist approaches to science and politics — it is both possible and useful for us to approach the study of genetics and the search for ‘gay genes’ from a queer perspective. Queer theorists have attempted, since the early 1990s, to negotiate a discursive space different from, but contiguous with, lesbian and gay studies (de Lauretis 1991, iv). Queer, in the formulation of theorists such as Michael Warner, is a social process and a negotiation of normativities, rather than an exploration of particular sexual identities (Warner 1993, xiii). While it is an open question how exactly we might define ‘queer’, and to what this term can or should be applied, I will here adopt Jagose's (1996) definition, which suggests that ‘queer describes those gestures or analytical models that dramatise incoherencies in the allegedly stable relations between chromosomal sex, gender and sexual desire’. Jagose adds to this that queer has ‘a conceptually unique potential as a necessarily unfixed site of engagement and contestation’, a view that is echoed by David Halperin when he claims that the word queer does not have a specific referent (1995, 62). I contend that these early statements about ‘queer’ fit well with recent work from feminist new materialist and ecological traditions, and that it allows us to turn the critical power of queer towards both the material reality of the biological and the body and their associated cultural meanings (Giffney and Hird 2008; see also Morton 2010). More specifically, I contend that queer demands a critical approach to the relations between sex and gender, nature and culture, the biological and the social, and thus is a useful tool for bringing critical realism, agential realism, and sexual science into conversation.

As noted, however, there are a number of tensions that result from engaging in such a project. For example, Karen Barad's agential realism has a fundamentally different approach to the subject/object distinction (as well as related distinctions between sex/gender, nature/culture) than approaches that are rooted in critical realism. In this article I do not aim to resolve these tensions but, rather, I aim to bring different knowledges into conversation with each other. This is what Barad describes as a ‘diffractive methodology’ (which she explicitly links to queer approaches), in which insights and approaches from different areas of study are read *through* each other (2007). I contend that work from feminist epistemologies of science, tools from agential realism, and concepts from critical realism can be brought into conversation with each other in this way in order to offer a very productive way of thinking about ‘gay genes’. This approach is useful, concretely, as it recognizes both the material reality of genes and the biological and social opportunities and constraints that they make possible, while stressing the fallible and situated nature of all knowledge claims about them as well. Before developing this argument in the final section of this article, however, the next few sections will explore the feminist approaches that will inform this queer understanding of genetics and gay genes. Specifically, these sections will begin by exploring the early feminist work of Sandra Harding, Nancy Hartsock and Donna Haraway, continue by discussing Karen Barad's agential realism, and conclude by examining Caroline New and Lena Gunnarsson's critical realist approaches.

## Queer conversations: three key resources

### Feminist epistemologies of science: Sandra Harding, Nancy Hartsock and Donna Haraway

Many early feminists engaging with science approached the issue of its relationship with politics by questioning the barriers and inequalities that resulted in more men than women entering the sciences. A concurrent and related feminist programme studied the use and abuse of biology, and argued that the key issue for feminism is the patriarchal use of science rather than science itself. Sandra Harding has helpfully described a series of the key feminist epistemologies that developed from these research programmes. In particular, she has described how these programmes have attempted to respond to an apparent paradox in feminist engagements with science, explained as follows:

Feminism is a political movement for social change. But many claims, clearly motivated by feminist concerns, made by researchers and theorists in the social sciences, in biology, and in the social studies of the natural sciences appear more plausible — more likely to be confirmed by evidence — than the beliefs they would replace. How can such politicized research be increasing the objectivity of inquiry? (Harding 1986, 24)

After all, the social identity of the scientist is meant to be irrelevant to the ‘goodness’ of the science. Certain feminist approaches have suggested, however, that ‘women (or feminists, whether men or women) *as a group* are more likely to produce unbiased and objective results than are men (or nonfeminists) as a group’ (Harding 1986, 25). Harding developed this claim into what are now commonly known as feminist standpoint epistemologies. These epistemologies suggest that scientific knowledge, when grounded in women's experience, produces ‘a morally and scientifically preferable grounding for our interpretations and explanations of nature and social life’ (Harding 1986, 26).

Relatedly, Nancy Hartsock sought to define a standpoint as ‘not simply an interested position (interpreted as bias) but … interested in the sense of being engaged’ (Hartsock 1983, 285). A feminist standpoint would therefore be able to counter the male ‘bias’ in the sciences, and any resultant female ‘bias’ should be interpreted not as a negative consequence but as part of a positive form of political engagement on the part of women. Donna Haraway, who has explicitly aimed to set her approach to science apart from relativism and radical social constructionism, has pursued a similar project. She explicitly aims to mediate between a position of social constructionism in science, on the one hand, and the political need for a feminist standpoint, on the other. She explains as follows:

I think my problem and ‘our’ problem is how to have *simultaneously* an account of radical historical contingency for all knowledge claims and knowing subjects … *and* a no-nonsense commitment to faithful accounts of a ‘real’ world. (Haraway 1991, 187, original emphasis)

Haraway's ‘situated knowledges’ approach therefore aims to account for both the contingent and constructed nature of scientific claims and the necessity of adopting a certain type of scientific realism for political projects. While it is not until her later work that Haraway began to explicitly associate her work with queer theory (1997, 2008) I contend that a situated knowledges approach can provide us with a number of resources for the development of a queer approach to gay genes. This is the case, specifically, because it critically negotiates the necessity of developing accounts of the real and the material while, at the same time, recognizing the radically contingent nature of all knowledge claims. Haraway suggests, for instance, that critically reducing the distance between subject and object will produce more faithful accounts of ‘reality’, even if the situated nature of such accounts of reality must always be recognized. One of the specific ways in which Haraway has suggested that this object-subject distinction can be broken down in order to encourage more contingent and partial connections, and thus more objectivity, is by emphasizing the agency of the object. She argues that there is a logic of domination built into traditional Western binaries such as object-subject, whereby one component side of the binary is always figured as passive and as a resource for the production or appropriation by the corresponding active component. Nature, for instance, is conceived of as the raw material or resource for cultural production, while sex is conceived of as a resource for the acts of gender, and the female is considered a resource for the male. As an alternative, Haraway emphasizes the agency of the object. She explains as follows:

Situated knowledges require that the object of knowledge be pictured as an actor and agent, not a screen or a ground or a resource, never finally as slave to the master that closes off the dialectic in his unique agency and authorship of ‘objective’ knowledge. (1991, 198)

Haraway argues that conceiving of objects as actors does not amount to a naïve form of scientific realism but, instead, involves an engagement with objects that depends on a ‘power-charged social relation of “conversation”’ (1991, 198). The fact that she takes this position arguably makes Haraway's approach a helpful resource for queering (gay) genes. Specifically, her approach prevents us from thinking about genes either as completely blank and waiting for cultural meanings to be inscribed upon them, or as master molecules dictating all of culture. Indeed, Haraway usefully insists that, ‘[t]he world neither speaks itself nor disappears in favour of a master decoder … The world is not raw material for humanization’ (1991, 198). This could be described as a queer approach because it emphasizes the material nature of biological sex and its relation to gender and sexuality while also critically destabilizing these seemingly stable relationships, particularly by means of its emphasis on the situated and fallible nature of all knowledge claims.

The next few sections will argue, however, that — in addition to the intellectual resources that Haraway, Harding and Hartsock can provide us with — both Karen Barad's articulation of an ‘agential realism’ and the critical realist feminist use of emergence are helpful if we are to queer (gay) genes, and truly do justice to the ‘critical’ in a potentially critical realist account of genes. I find Barad's concept of agential cuts particularly productive when thinking about gay genes. It is to a discussion of this element of her work, therefore, that the following sections will turn.

### Agential realism: Karen Barad

In employing Barad's agential realism it is important to begin by drawing attention to the fact that her approach relies to a significant extent on Niels Bohr's account of quantum physics. Bohr was a Nobel prize-winning physicist whose work was foundational for twentieth-century physics. In addition to this, however, it is also important to emphasize that Bohr's account is by no means the only available account of quantum mechanics. Indeed, critical realists such as Alistair Mutch have argued that Christopher Norris's use of David Bohm's hidden variables theory may be preferable to Barad's use of Bohr (Mutch 2013, 35). While my focus in this article will be on Barad's agential realism it is therefore worth remembering throughout that quantum physics is ‘open and contested’ (Mutch 2013, 35), both in terms of its formulation by physicists and in terms of the range of subjects to which it can be applied or brought into conversation with. Indeed, I agree with Susan V. Scott and Wanda J. Orlikowski's assessment when they state that accepting ‘multiple views of quantum physics as instances of debate’ and embracing ‘open dialogue across different perspectives’ is of key importance (2013, 79).

Notwithstanding such qualifications, I continue to find Barad's agential realism instructive. This is the case, in particular, because I think that her approach has significant queer potential. Barad herself has, in fact, described the approach that is developed in her work as ‘an account of technoscientific and other practices that takes feminist, antiracist, poststructuralist, queer, Marxist, science studies, and scientific insights seriously’ (2003, 810–11). Indeed, she explicitly and repeatedly articulates agential realism as a queer approach. I find this use of ‘queer’ in relation to quantum mechanics suggestive, and find it productive when it comes to my attempt to think of genes as real active agents that emerge from complex co-acting biological and social contexts without relying on strict dichotomies such as sex/gender, male/female and social/natural. Indeed, I find Barad's work productive for the development of a queer approach that is committed to troubling the seemingly stable relations between sex, gender and sexuality, while also being committed to the real material nature of biology, particularly genes.

A central element of Barad's agential realism concerns Bohr's disagreement with physicist Werner Heisenberg over the uncertainty principle. This principle states that the act of measurement affects the premeasurement values of the particle being measured: the more precisely the position is measured, the less precisely the momentum can be measured (and vice versa). Bohr, on the other hand, argued that the act of measurement actually causes the properties of the particle (such as position and momentum) to become determinate, governed by the specificity of the experimental apparatus (Barad 2007, 19). Barad summarizes their different approaches:

The nature of the difference between their views … can be summarized as follows: For Bohr, what is at issue is *not* that we cannot *know* both the position and momentum of a particle simultaneously (as Heisenberg initially argued), but rather that particles do not *have* determinate values of position and momentum simultaneously. (Barad 2007, 19, original emphasis)

Barad draws on the latter of these approaches in order to develop her own agential realist approach. This approach insists that ‘the primary ontological unit is not independent objects with inherent boundaries and properties but rather *phenomena*’ (Barad 2007, 139, original emphasis). She offers the following definition:

‘Phenomena’, in an agential realist sense, are the entanglement … of intra-acting agencies. (Where agency is an enactment, not something someone has, or something instantiated in the form of an individual agent.) It is through specific agential intra-actions that the boundaries and properties of ‘individuals’ within the phenomenon become determinate and particular material articulations of the world become meaningful. (Barad 2012, 77)

For Barad, objects do not have extra-discursive properties; rather, properties come into existence through the emergence of phenomena in relations of observation and measurement. Importantly, phenomena are:

produced through complex agential intra-actions of multiple material-discursive practices or apparatuses of bodily production, where *apparatuses are not mere observing instruments but boundary-drawing practices — specific material (re)configurings of the world — which come to matter.* (Barad 2007, 140, original emphasis)

While Barad's account of phenomena may seem incompatible with certain claims in the critical realist tradition, thinking of genes as phenomena in this manner provides us with a way of thinking through the fact that the word ‘gene’ refers to different biological processes or entities across different sub-disciplines of biological science. From an agential realist perspective this is the case because the gene is ‘produced’ — or emerges — through certain material-discursive reconfigurings of the world or apparatuses. Importantly, however, the fact that different apparatuses in different disciplines allow for a different gene to emerge does not take away from the reality of the gene.

Particularly key for Barad's articulation, and for my attempt to both queer the search for gay genes and look for the queer in the genes themselves, is her concept of ‘agential cuts’. This concept refers to the boundary-making practices that allow phenomena to emerge in complex situated material-discursive contexts. Barad argues that:

It is through specific agential intra-actions that the boundaries and properties of the components of phenomena become determinate and that particular concepts (that is, particular material articulations of the world) become meaningful. Intra-actions include the larger material arrangement (i.e., set of material practices) that effects an *agential cut* between ‘subject’ and ‘object’ … That is, the agential cut enacts a resolution *within* the phenomenon of the inherent ontological (and semantic) indeterminacy. In other words, relata do not preexist relations; rather, relata-within-phenomena emerge through specific intra-actions. (Barad 2007, 139–40, original emphasis)

Barad's agential realism therefore claims that ontology and epistemology do not refer to an extra-discursive reality, but that they are only made separate by particular agential cuts — cuts, that is, which are historically and socially situated. At a broad meta-theoretical level, this claim seems to provide a helpful answer to Haraway's call for reducing the subject/object divide. Indeed, it arguably paves the way for us to pay much greater attention to the agency of objects, and encourages the development of knowledge by means of conversation and partial connections. At a more specific level, however, we can see a clear example of an agential cut within the gay genes research that was discussed above, in the cut that produces the categories ‘homosexual’ and ‘heterosexual’. In Bailey and Pillard's research, for instance, participants were asked to score themselves along the Kinsey scale, and — because this scale measures sexuality in a non-dichotomous way — they therefore did not have to categorize themselves according to the binary of heterosexual/homosexual. For the purposes of data analysis, however, both exclusively homosexual participants and those considered ‘substantially bisexual’ were combined to produce the 52 per cent of monozygotic twins considered to be homosexual (Stein 1991, 94). Bailey and Pillard therefore enacted a series of agential cuts in order to form the two categories: homosexual and heterosexual. These are not, however, extra-discursive natural kinds but, rather, are formed through measurement, intervention, and the enactment of particular cuts in particular times and places. Hamer's insistence that ‘one form of homosexuality’ is genetically linked to the chromosomal region that he studied also begs various questions related to the making of agential cuts. For instance, which ‘form’ of homosexuality is it that is linked in this manner? Indeed, how exactly is it that supposedly distinctive forms of sexuality are distinguished from each other, whether genetically or socially?

### Critical realism: Caroline New and Lena Gunnarsson

Critical realism approaches the distinction between subject and object and between epistemology and ontology differently than does Barad's agential realism. In much of the critical realist literature, for instance, a distinction between the transitive and the intransitive dimensions of knowledge is strictly maintained. In this way the changing nature of knowledge about the world (the transitive dimension) and the relatively stable ontological reality of the world itself (the intransitive dimension) are held to be distinct. Instead, Barad's approach, while it certainly accepts the transitive nature of scientific knowledge and the ontological reality of the world, asks critical questions about where the line is to be drawn (or the cut is made) between these two seemingly separate dimensions. I do not mean to attempt to resolve this tension here; rather, I hope to use tools from both critical realism and agential realism to think about gay genes from within a queer framework. To that end, I will discuss the critical realist concepts of generative mechanisms and emergence in this section, particularly as they are employed by Caroline New and Lena Gunnarsson in their attempts to articulate a critical realist approach to sex, gender and sexuality.

Caroline New develops a critical realist approach to gender in response to, and somewhat in opposition to, the feminist standpoint and situated knowledges approaches that were explored earlier. New argues that elements of both Harding's approach and Haraway's situated knowledges approach could be considered forms of critical realism. For example, New's argument that ‘the world is *already* differentiated, complex and stratified, in ways which our concepts may or may not adequately express’ (2005, 56–7, original emphasis) appears to resonate with Haraway's insistence that, ‘[t]he world neither speaks itself nor disappears in favour of a master decoder … The world is not raw material for humanization’ (1991, 198). New links this always-already complex ontology of the world to the critical realist concept of generative mechanisms, which she also describes as ‘causal configurations’ or ‘causal powers’ (2005, 58). Andrew Collier (1994) has described such generative mechanisms as always multiple and active — co-creating structures, contexts and events. I find this concept useful, particularly when it is linked to Haraway's concept of situated knowledges. This is the case because mechanisms need not just be scientific discoveries, procedures or economic funding into particularly scientific endeavours. Usefully for my argument, mechanisms are not so mechanistic, and can involve the complex co-acting paradigms, histories, and social and political contexts that are sometimes ignored in conventional histories of scientific progress. For instance, by using critical realist language it becomes possible to describe the Human Genome Project as one of the several co-acting generative mechanisms that has been at work within the broader context of research into ‘gay genes’. This is useful because Hamer's aforementioned study is explicit about the fact that advances in genome mapping techniques were generative in providing the possibility for his research. Of course, these techniques were in turn made possible by the enormous amount of multi-national funding that went into developing the Human Genome Project.

New's approach to sex and gender attempts to formulate the relationship between these terms as one of emergence and non-dichotomous distinction. However, it is possible to read her account as one in which nature tends to become a resource for culture just as sex is a resource for gender, which is problematically close to the female being a resource for the male. From this perspective, New's critical realist approach is useful but, perhaps, not sufficiently critical. New does, however, anticipate some of these objections when she quotes the work of Rosi Braidotti, who has argued that ‘as soon as the categories of the social and the natural are dichotomised, the sexes likewise are polarised in a situation of dialectical confrontation’ (1994, 129). I agree with this assessment, and therefore suggest that New's approach is perhaps not *queer* enough. That is to say, it depends to too large an extent on a number of very strict dichotomies, particularly those of sex/gender, male/female and the social/the natural. For instance, her account explicitly argues that a critical realist account of sex and gender leads to the conclusion that not only is biological sex ‘real’, but that it only *really* comes in two forms, male and female. Evidence to the contrary, such as scholarship on intersex, is therefore dismissed or neglected (New 2005, 63).

Lena Gunnarsson's work shows, however, that the adoption of a realist position does not need to result in the making of such assumptions about sexual dimorphism. When she argues that men and women are differently positioned as men and as women, for instance, Gunnarsson is clear that she does not mean that ‘all people are either men or women in any unambiguous sense’ (2011, 35 n. 8). While Gunnarsson does explicitly support New's position on sex and gender elsewhere (2014), her work may be used to provide a more nuanced argument than the work of New. In particular, I see queer potential in her recognition of ambiguity — specifically, there is potential for the development of a critical account that recognizes the material reality of biological sex, but can still do some of the queer work that is required in order to destabilize the supposedly strict and stable relations between sex, gender and sexuality. In addition to her arguments about intersex, however, I also find Gunnarsson's account more convincing because of her careful elaboration of the concept of emergence. Gunnarsson explains emergence within critical realism as referring to the idea that ‘something, here and now, is composed by the powers and properties of something else while still possessing its own unique powers and properties’ (Gunnarsson 2013, 15). In this way, gender is said to *emerge* from biological sex, without being fully determined or predicted by this, and without necessarily having to depend upon dimorphism in terms of biological sex. Certainly, New's account is also an attempt at developing such an emergentist approach, but some of the agential cuts that she makes in order to insist on the dimorphism of biological sexual difference are arguably rooted in lay assumptions rather than ontological truths.

Queer, as I have been employing it thus far, draws attention to the need to account for the reality of sexual difference, while simultaneously recognizing the always historically contingent nature of such differences and the fallibility of all knowledge claims about them. This continues to be an important point to make because of the enduring influence of naïve scientific realist approaches that develop biological determinist accounts of sexual difference. Such approaches suggest that individuals have natural biological characteristics or properties that can be observed, measured, and then used to classify those individuals. Indeed, these approaches suggest that, whether at the level of bodies, organs, genes, hormones or chromosomes, differences between individuals are best accounted for by means of biological explanations. In this account, representations of sexual difference are reflections of the natural, pre-social and pre-intervention (such as measurement or classification) properties of individuals. Social constructivist accounts, on the other hand, have embraced the opposite position, and have suggested that representations of sexual difference are not reflections of any ‘true’ nature, but should be understood as cultural constructions. As opposed to both of these perspectives, a critical realist account might suggest that the social emerges from the biological, but that it is not reducible to it. However, this argument may, at least in some of its manifestations, hinge on certain lay assumptions about biology and ontology. As critical realism stresses the fallibility of all knowledge, however, I contend that a form of vigilance is required with regard to these lay assumptions.

The adoption of a more modest approach by proponents of the critical realist approach, importantly, would mean that it fits in well with the development of a queer account. This is the case because such an approach attempts to take seriously the material nature of genes (along with their real material effects on bodies, behaviours and communities) while also insisting that these effects are not deterministic, that they always emerge in particular historical and cultural contexts, and that knowledge claims are always fallible and socially and historically situated. Queer contains within itself a commitment to the possibility of radical revision as well, and is always open to change and reconfiguration.

## Conclusion: queer(ing) genes

To attempt to approach genetics and the search for gay genes from a queer perspective — to queer genes — is therefore to attempt multiple negotiations. Throughout this article, it has been my argument that queer is useful for negotiating the often-oppositional relationship of the biological and the social, and I have sought to emphasize both the real material nature of sex, gender and sexuality and the fallibility and situatedness of all knowledge claims in this area. This relates, as we have seen, to ongoing debates within scientific research into sexuality — in particular, it relates to the question of whether homosexuality is biological or social, usually expressed in terms of ‘born or made’. Framing the debate about sexuality in such terms, however, offers us only two alternatives. By drawing on the aforementioned three intellectual resources, it becomes clear that this is in fact a false choice. Feminist standpoint epistemologies, for instance, emphasize the political nature of scientific knowledge. Haraway's situated knowledges framework is committed both to the real *and* to the fallibility of all knowledge claims. Critical realism adds to this the idea that the biological and the social are not, in fact, in opposition, but are instead rooted in a relationship of emergence. Finally, an agential realist account provides us with the concept of agential cuts, which is useful for stressing the fact that differences and boundaries are always socially and historically contingent. Indeed, this approach suggests that these differences and boundary-making practices are necessarily open to debate and reconfiguration, with new cuts creating new and different boundaries. Approaches such as these, then, are able to help us maintain the importance of material reality while also emphasizing the situated natures of the agential cuts that are necessary in order to produce knowledge.

Bringing these different approaches into dialogue does, however, lead to a number of tensions, including those relating to the epistemic fallacy. As an idea, the epistemic fallacy depends upon the separation of ontology and epistemology, and refers to the reduction of the former to the latter. Jeroen Van Bouwel suggests, however, that unveiling the epistemic fallacy at work often leads to committing what he calls the ontological fallacy. This refers to situations in which ‘taking an a priori ontological stance … transposes or reduces epistemological and methodological matters into an ontological matter’ (2003, 85). For Van Bouwel, the separation between ontology and epistemology is important, and he maintains that both of the aforementioned fallacies fail to uphold the distinction. Barad's account, instead, very much depends on the *inseparability* of the ontological and the epistemological. I do not wish to argue that they are inseparable in this way (Gunnarsson 2015). Rather, I simply claim that the boundary-making practices between ontology and epistemology are contingent, historical and cultural, and that they are themselves open to debate and reconfiguration. In this regard, Michael S. Carolan emphasizes that paying attention to the epistemic fallacy requires that we accept the fact that all knowledge claims are fallible. Indeed, he argues that the fallibility of all knowledge claims ‘is the crux of critical realism (and, thus, what makes critical realism critical)’ and, crucially, that this opens ‘knowledge claims to criticism, testing, and further improvement’ (2005, 396). If, following Carolan, we agree on the fallibility of all knowledge claims, however, we must also ask ourselves whether this includes the knowledge of where to draw the line between ontology and epistemology.

Despite such tensions, it seems clear that agential realism and critical realism can be brought into conversation with each other in interesting ways. Tools and resources from both of these approaches can illuminate some of the ambiguities that are inherent to the search for gay genes. For example, the social ‘gene’ may emerge from the biological gene, but this biological gene itself emerges from a number of complex co-acting generative mechanisms. Not only this, but the gene emerges differently in different sub-disciplines of biological science, due to the making of agential cuts and the different material-discursive configurations of the apparatuses at work within different areas of scientific knowledge production. This is not to say that human beings ‘create’ the gene, but that genes become real in this sense through specific material-discursive arrangements of the agency of the world. As discussed earlier, Halperin has suggested that the word ‘queer’ does not have a single specific referent (1995, 62). I therefore conclude by proposing that a possible adjective for genes could be queer. In such a formulation, Halperin's unassuming word and Johannsen's applicable little word combine to suggest irreducibly complex queer genes. These genes are not structural biological units; instead, ‘queer genes’ refers to the complex agential reality of genes, both in their biological and their social meaning. This draws attention to the strangeness and surprising nature of genetic processes, something that has been illustrated by the Human Genome Project. It also stresses the real material constraints that genes impose upon humans as biological-social organisms, while allowing space for the opportunities and possibilities that they enable. This queer approach, drawing on feminist science studies, agential realism and critical realism, can emphasize the real material nature of genes and the effects they have on bodies, behaviours and communities. Importantly, however, it can also do some of the queer work of critically negotiating the boundary between the biological and the social and challenging the seemingly stable relations between sex, gender and sexuality.

## Notes

1 There have been a number of subsequent studies into genetic bases for homosexuality, with mixed results (see, for example, Hu et al. 1995; Sanders et al. 1998; Bailey et al. 1999; Rice et al. 1999; Mustanski et al. 2005; Ramagopalan et al. 2010; Sanders et al. 2014).

2 The metaphor of mapping did not lose its appeal as the project continued. When the Human Genome Project published its ‘first draft’ in 2000, announced simultaneously by US President Bill Clinton and UK Prime Minister Tony Blair on 26 June, Clinton described the draft as ‘without a doubt … the most important, most wondrous map ever produced by humankind’ (Kukis 2000).

3 Bailey also authored one of the rarer studies of the time on female homosexuality (Bailey and Benishay 1993).

4 Previous research on the maternal nature of inherited homosexuality include Henry (1941), Zuger (1989) and Pillard and Weinrich (1986).

5 See note 1.
